# Teclistamab in relapsed refractory multiple myeloma: multi-institutional real-world study

**DOI:** 10.1038/s41408-024-01003-z

**Published:** 2024-03-05

**Authors:** Meera Mohan, Jorge Monge, Nishi Shah, Danny Luan, Mark Forsberg, Vineel Bhatlapenumarthi, Metodi Balev, Anannya Patwari, Heloise Cheruvalath, Divaya Bhutani, Sharmilan Thanendrarajan, Binod Dhakal, Maurizio Zangari, Samer Al-Hadidi, Dennis Cooper, Suzanne Lentzsch, Frits van Rhee, Anita D’Souza, Aniko Szabo, Carolina Schinke, Rajshekhar Chakraborty

**Affiliations:** 1 Division of Hematology/Oncology, Department of Medicine, Medical College of Wisconsin, Milwaukee, WI USA; 2 Division of Hematology/Oncology, Weill Cornell Medicine, New York, NY USA; 3 Division of Hematological Malignancies, Department of Oncology, Montefiore Medical Center and Albert Einstein College of Medicine, New York, NY USA; 4 Multiple Myeloma and Amyloidosis Program, Columbia University, Herbert Irving Comprehensive Cancer Center, New York, NY USA; 5 Medical College of Wisconsin Medical School, Milwaukee, WI USA; 6 Myeloma Center, University of Arkansas for Medical Science, Little Rock, AR USA; 7 Division of Biostatistics, Institute of Health and Equity, Medical College of Wisconsin, Milwaukee, WI USA

**Keywords:** Myeloma, Drug development

## Abstract

The objective of our study was to report real-world data on the safety and efficacy of standard-of-care teclistamab in patients with relapsed/refractory multiple myeloma (MM). This is a multi-institutional retrospective cohort study and included all consecutive patients that received at least one dose of teclistamab up until August 2023. One hundred and ten patients were included, of whom, 86% had triple-class refractory disease, 76% penta-refractory disease, and 35% had prior exposure to B-cell maturation antigen (BCMA)-targeting therapies. The overall response rate (ORR) in our cohort was 62%, with a ≥ very good partial remission (VGPR) rate of 51%. The ORR in patients with and without prior BCMA-targeted therapies was 54% vs 67%, respectively (*p* = 0.23). At a median follow-up of 3.5 months (range, 0.39–10.92), the estimated 3 month and 6 month progression free survival (PFS) was 57% (95% CI, 48%, 68%) and 52% (95% CI, 42%, 64%) respectively. The incidence of cytokine release syndrome (CRS) and immune effector cell associated neurotoxicity syndrome (ICANS) was 56% and 11% respectively, with grade ≥3 CRS and ICANS noted in 3.5% and 4.6% of patients respectively. 78 unique infections were diagnosed in 44 patients, with the incidence of all-grade and grade ≥3 infections being 40% vs 26% respectively. Primary prophylaxis with intravenous immunoglobulin (IVIG) was associated with a significantly lower infection risk on multivariate analysis (Hazard ratio [HR] 0.33; 95% CI 0.17, 0.64*; p* = 0.001).

## Introduction

T-cell redirecting immunotherapies including bispecific antibodies (bsAbs) and chimeric antigen receptor T-cells (CAR T) therapy have transformed the treatment of relapsed/refractory multiple myeloma (MM) [1–6]. Notably, bsAb are off-the-shelf therapeutic options readily available to patients with aggressive disease relapse, with a lower incidence of severe acute toxicities such as cytokine release syndrome (CRS) or neurotoxicity, and potential ability to deliver treatment in the community, which can improve access to underserved populations [7]. Currently, two bsAbs targeting B-cell maturation antigen (BCMA) have received accelerated approval by the FDA-teclistamab and elranatamab [1, 6]. Teclistamab was the first BCMA-targeted bsAb to receive accelerated approval in November 2022 based on a single-agent overall response rate (ORR) of 63% and a median duration of response (DoR) of 18.4 months in a heavily pre-treated population [1]. In a recent update, with a follow up of ~ 2 years, 43% of patients achieved a ≥CR with a median PFS of 12.5 months and a median DOR of 24 months [2].

A critical gap still remains regarding data on safety and efficacy of standard-of-care teclistamab since its approval in the US. Real-world data on safety and efficacy of teclistamab is critically important due to two reasons: First, the strict inclusion and exclusion criteria of clinical trials eliminated patients with co-morbidities who might be at a higher risk of severe or fatal toxicity from teclistamab. For example, patients with creatinine clearance less than 40 ml/min or absolute neutrophil count (ANC) less than 1000/cc were excluded from the MajesTEC-1 trial [1]. High grade infection and infection-related mortality has emerged as an important toxicity signal with BCMA-targeting bsAbs [1, 6, 8, 9]. Notably, infection prophylaxis was not standardized in the seminal clinical trials that led to the approval of bsAb in MM. Currently, infection prophylaxis may be used more commonly in routine clinical practice compared to clinical trials since the signal for severe infection-related morbidity and mortality is now well recognized. Second, the prolonged screening period of early-phase clinical trials may inherently eliminate some patients with aggressive relapse who need immediate treatment initiation, leaving a knowledge gap that only pragmatic trials or real-world data can fill. Moreover, the real-world safety and efficacy of teclistamab following relapse from BCMA CAR T-cell therapy remains largely unknown [1, 6]. This brings us to the key objective of our multicenter retrospective cohort study, i.e., to assess the efficacy and safety of standard-of-care single-agent teclistamab representing a diverse MM patient population across 5 academic U.S. medical centers.

## Subjects and methods

### Patients and clinical end points

Patients in this study had relapsed and/or refractory MM and were treated with teclistamab as standard of care at one of 5 academic centers in the US-Medical College of Wisconsin, Milwaukee, WI, University of Arkansas for Medical Sciences, Little Rock, AR, Columbia University Irving Medical Center, New York, NY, Montefiore Medical Center and Albert Einstein College of Medicine, Bronx, NY, and Weill Cornell Medical College, Cornell University, New York, NY. Patients who received at least one dose of teclistamab were included. Data including patient demographics, disease characteristics (including bone marrow biopsy, fluorescence in situ hybridization studies [FISH], and baseline advanced imaging with positron emission tomography – computed tomography [PET-CT] or magnetic resonance imaging (MRI) at the most recent assessment before starting treatment) were collected. Triple class refractory (TCR) disease was defined as refractory to anti-CD38 monoclonal antibodies, proteasome inhibitor (PI) and immunomodulatory agents (IMiD) [10]. Penta drug refractory MM was defined as disease refractory to daratumumab, bortezomib, carfilzomib, lenalidomide and pomalidomide. High risk disease was defined as the presence of t(4;14); t(14;16); t(14;20); 1q21 copy number abnormalities, or deletion (17 p) by FISH. Extramedullary (EMD) disease was defined as plasma cell clone growing outside the bone marrow without any anatomical connection to the bone [11].

Data on serum immunoglobulin levels and blood counts were obtained at baseline prior to starting treatment. Serial immunoglobulin (IgG, IgA, IgM) levels were also collected at day 1 of every cycle of treatment protocol or per institutional practice. Hypogammaglobinemia was defined as IgG levels of ≤ 400 mg/dL. The functional IgG was computed by subtracting serum M-protein values from IgG levels in IgG subtype MM. Neutropenia was defined as absolute neutrophil count of ≤1 × 10^3^/µl and lymphopenia as an absolute lymphocyte count of ≤1 × 10^3^/µl.

Patients received a step-up dose of teclistamab at 0.06 mg/kg, 0.3 mg/kg, and 1.5 mg/kg on either a D1, D4, D7 or D1, D3, D5 schedule per individual institutional guidelines. Step up dosing were administered either inpatient or outpatient per institutional practices. Subsequent doses were given either at 1.5 mg/kg weekly or alternative schedule at the physician’s discretion. CRS and immune effector cell associated neurotoxicity syndrome (ICANS) were graded according to the American Society for Transplantation and Cellular Therapy consensus [12]. Management of CRS and ICANS were done according to institutional guidelines.

Supportive care, infections prevention and monitoring strategies were implemented according to institutional guidelines. Primary intravenous immunoglobulin (IVIG) prophylaxis refers to preemptive IVIG replacement in patients with hypogammaglobulinemia, irrespective of any history of infection. Infections confirmed by clinical, imaging, microbiological, or histopathological evidence were captured from day 1 of the first cycle of bsAb until the last follow-up or 60 days after completion of therapy. Since continuous bsAb therapy can lead to depletion of endogenous plasma cells including long lived plasma cells and possible T cell exhaustion, we collected infection data for up until 60 days after last dose of teclistamab. The National Cancer Institute Common Terminology Criteria for Adverse Events, version 5, was used to describe the site and degree of infections.

The study was approved by the Institutional Review Board of the coordinating institution (Medical College of Wisconsin) and subsequently by all participating institutions. The research was performed in compliance with the terms of the declaration of Helsinki. Data cutoff was August 31st, 2023.

### Statistical analysis

Demographic and disease characteristics of the study population were summarized using counts with percentages for categorical variables and median with range for continuous measures. The overall response rate was defined as the proportion of patients achieving PR, VGPR, or CR at any time, with time to best response computed as the time from the start of the treatment to the first documented evidence of the best achieved response. Logistic regression was used to evaluate predictors of response.

Overall and progression-free survival were estimated using the Kaplan-Meier estimator with 95% confidence intervals based on the log-log transform. The cumulative incidence of infection was estimated using the Nelson-Aalen estimator with death without infection as competing risk. Gray’s test was used to compare the cumulative incidence between patient groups. Infection density was estimated as the number of infections divided by the total days at risk, with inference based on person-level quasi-Poisson regression of the number of infections with the log-transformed number of days at risk as offset. Selected variables of interest such as serial immunoglobulin and blood count variables i.e lymphocyte and neutrophil counts were summarized over the course of treatment by selecting the earliest available measurement within each range of teclistamab cycles (1–4, 5–9, etc). These were then compared to the baseline counts for the same subjects using a paired test. The risk factors for infections were modeled using a day-level Cox regression model with robust standard errors to account for within-subject repetitions. This model was selected as it allows the inclusion of recurrent infections and both baseline and time-varying covariates.

## Results

A total of 110 patients received teclistamab between January 2023 and August 2023. A detailed description of baseline characteristics of patients can be found in Supplement Table 1. The median age was 68 (range 37–89) years with 45 (41%) patients ≥70 years and 28 (25%) ≥75 years of age. Females accounted for 49% (*n* = 54) of patients included in this study. About 29% (*n* = 32) of patients were African American, 10% Hispanic (*n* = 11) and 1.8% (*n* = 2) were Asian or Pacific Islander. The most prevalent heavy chain subtype was IgG (51%; *n* = 54) and the most common light chain involved was kappa (70%; *n* = 77). High-risk FISH abnormalities were present in 62% (*n* = 59) patients, and 44% (*n* = 48) had EMD. Two patients included in this study had plasma cell leukemia (PCL) and 3 patients had concomitant underlying immunoglobulin light chain (AL) amyloidosis. The median prior lines of therapy were 6 (range 3–13). The majority had triple class refractory disease (86%; *n* = 95) and 76% (*n* = 84) had penta refractory MM. In the current study, 35% (*n* = 38) patients received prior BCMA targeted therapy, including 18 patients who received a prior BCMA CAR T-cell therapy. Twelve patients had received belantamab mafadotin and 2 with both prior BCMA CAR T-cell therapy and belantamab mafadotin before teclistamab therapy. Additionally, 5 patients had received investigational BCMA bsAb therapy. The majority of patients (94%, *n* = 103) had progressive disease at the time of initiation of teclistamab therapy. A total of 87% of patients has at least one autologous stem cell transplant (ASCT) and 2.7% (*n* = 3) had a prior allogenic transplant. At the start of teclistmab therapy, the median absolute neutrophil count and absolute lymphocyte counts were 2.7 × 10^3^/µl and 0.82 × 10^3^/µl, respectively. In this cohort, 5.5% (*n* = 6) of patients were neutropenic and 59%(*n* = 65) were lymphopenic at the start of teclistamab therapy. At baseline, IgG, IgM, and IgA levels were 637 (range 338–2048) mg/dL, 11(6–25) mg/dL and 18 (range 7–110) mg/dL respectively. Further, the median functional IgG levels at baseline was 504 (range 273–762) mg/dL. Overall, at baseline, 32% (*n* = 32) of patients had hypogammaglobinemia and 39% (*n* = 37) functional hypogammaglobinemia.

While majority of patients received step-up doses of teclistamab as an inpatient, 10 (9%) patients underwent entirely outpatient treatment. As a comparison, we also assessed the baseline characteristics of the patients included in this analysis against those in the pivotal MajesTEC 1 clinical trial (Table 1) [1].

**Table 1:** Comparison Between the Real World and MajesTEC-1 Study Population.

Characteristic	Real World Study (N = 110)	MajesTEC 1 (N = 165)
Age; median (range)-years	68 (range 37-89)	64 (range 33-84)
≥75 yr.-no. (%)	28 (25)	24 (14.5)
Female -no. (%)	54 (49)	69 (41.8)
Race- no. (%)		
White	67 (61)	134 (81.2)
Black	32 (29)	21 (12.7)
Hispanic	11 (10)	NA
Asian	2 (1.8)	3 (1.8)
Plasma cell leukemia no. (%)	2 (1.8)	NA
Concomitant AL amyloidosis no. (%)	3 (2.7)	NA
Previous autologous stem cell transplantation- no. (%)	96 (87)	135 (81.8)
Previous therapy exposure- no. (%)		
Triple class refractory disease	95 (86)	128 (77.6)
Penta drug refractory disease	84 (76)	50 (30.3)
Median prior lines of therapy (range)	6 (range 3-13)	5 (range 2-14)
Prior exposed to BCMA directed therapy- no. (%)	38 (35)	0
High risk cytogenetic profile no./total no. (%)	59//95 (62) ^a^	38/148 (25.7)^b^
Extramedullary disease no. (%)	48 (44)	28 (17) ^c^
Median follow up (months)	3.5	22
CRS [all grades/ ≥ grade 3] -%	56/3.6	72/0.6
ICANS [all grades/ ≥ grade 3] -%	11/4.5	14/0.6
Infections [all grades/ ≥ grade 3] -%	40/26	76.4/44.8
ORR -%	62	63
≥VGPR -%	51	58.8

*CRS* cytokine release syndrome; *ICANS* immune effector cell associated neurotoxicity syndrome, *ORR* overall response rate, *VGPR* very good partial remission; ^a^1q21gain/amp, del(17p), t(4;14) and t(14;16), ^b^del(17p), t(4;14), t(14;16); ^c^presence of ≥1 extramedullary plasmacytoma.

### Efficacy

**Fig. 1:**
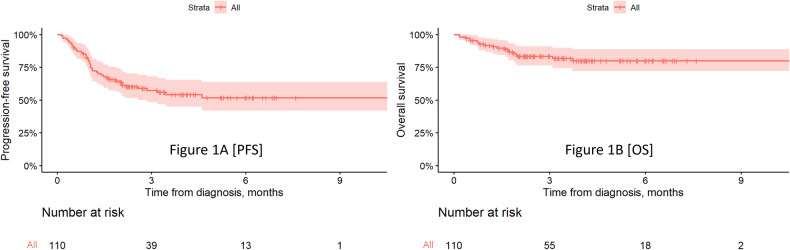
Kaplan-Meier Curves for Progression-Free Survival (PFS) and Overall Survival (OS) in the Study Cohort. **A** PFS; (**B**) OS.

At a median follow up of 3.5 (range 0.4–10.9) months, the ORR was 62% (*n* = 61) among the 98 evaluable patients. The proportion of patients achieving a VGPR or better ( ≥ VGPR) was 51%, with 20% of patients achieving a CR thus far. The ORR were not statistically different in patients with high-risk disease (44% vs 56%; *p* = 0.42), EMD (43% vs 58%; *p* = 0.46) and recipients of prior BCMA directed therapy (46% vs 54%; *p* = 0.23) (supplement table 2). In evaluable patients, the median time to best response was 1.67 (range 0.2–5.9) months. The median PFS and OS were not reached in this study. The 6 month PFS and OS were 52% (95% CI:42–64%) and 80% (95% CI: 72–89%), respectively (Fig. 1). Teclistamab was additionally given to patients with PCL (*n* = 2) and AL amyloidosis (*n* = 3) in our study. After the first full dose of teclistamab, both patients with secondary PCL experienced fulminant disease relapse. Notably, among the 3 patients with AL amyloidosis that were treated with teclistamab, all achieved ≥VGPR, and one patient achieved cardiac and renal organ response. At latest follow-up, one patient with AL amyloidosis had died 40 days after the last teclistamab dose due to progressive cardiac deterioration despite achieving hematological VGPR. We also observed a case of tumor pseudoprogression with spinal cord compression in a heavily pre-treated patient with early biochemical response following the first full dose of teclistamab.

Next, we analyzed factors predicting response to teclistamab therapy. Specifically, age, high-risk disease, presence of EMD, baseline lymphocyte count, prior lines of therapy and prior exposure to BCMA directed therapy was not significantly associated with response to treatment.

### Safety

Initial step-up doses were administered entirely outpatient in 9 patients in this cohort. Of the patients treated as an outpatient, 7 experienced CRS including grade 2 CRS in 1 patient. CRS was managed entirely outpatient except for 1 patient who was admitted for the management of CRS. Majority of patients (*n* = 101; 91%) received step up doses inpatient. For the entire cohort, the median duration of inpatient stay was 9 (range 0–75) days with 29% (*n* = 30) of patients requiring re-admission within 30 days of starting teclistamab therapy. The most frequent cause for readmission was infectious complications.

About 56% of patients had CRS to teclistamab therapy, with 93% of these being grade 1 or 2 CRS. Grade 3 CRS was observed in 1 patient and grade 4 CRS in 4 patients. ICANS was noted in 11%(*n* = 12) of patients in this study. ICANS of ≥ grade 3 was noted in 5 patients (4.5%) with 1 grade 5. The grade 5 event occurred in a heavily pretreated patient who developed steroid refractory ICANS and hemophagocytic lymphohistiocytosis syndrome after the first full dose of teclistamab. About a third (36%; *n* = 40) of patients received tocilizumab for CRS. Systemic steroids for CRS and/or ICANS was administered in 17% (*n* = 19) patients.

### Prophylaxis and infection characteristics

In our study cohort, infectious disease prophylaxis measures were as follows: prophylaxis for herpes simplex virus/varicella zoster virus (HSV/VZV) was used in 99% (*n* = 108) of patients, primary pneumocystis jiroveci prophylaxis in 57% (*n* = 61) patients, and primary anti-microbial prophylaxis was implemented in 12% (*n* = 13) of patients. About 14% (*n* = 15) of patients in this study received anti-fungal prophylaxis. Primary IVIG prophylaxis was employed in 46 (43%) patients.

**Fig. 2:**
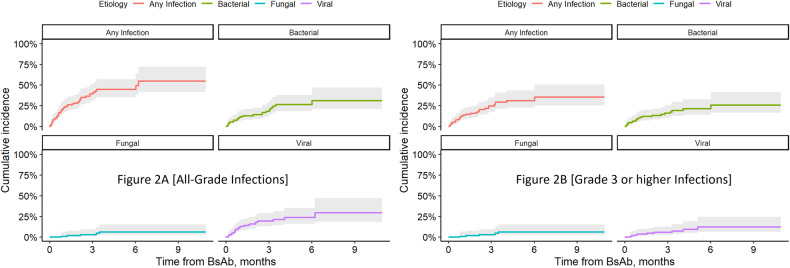
Kaplan-Meier curves for cumulative incidence of infections with teclistamab. **A** Cumulative incidence of all-grade infections. **B** Cumulative incidence of grade 3 or higher infections.

A total of 78 infections were diagnosed in 44 patients. The incidence of all grades and ≥ grade 3 infections was 40%(*n* = 44) and 26% (*n* = 29) respectively, with the 3 month and 6 month cumulative risk for any infection being 39% (95% CI, 31–51) and 45% (95% CI, 35–57) respectively (Fig. 2). The cumulative incidence of grade 3 or higher infections at 3 months and 6 months was 25% and 31% respectively (Fig. 2). The rates of any infection per 100 days in this cohort was 0.68.

The median time to first infectious event was 60 (range 23–99) days from the start of teclistamab therapy. The most common etiology was bacterial (48%; 36), followed by viral (*n* = 34; 45%) and fungal (*n* = 5; 6.7%). Infections were confirmed both clinically and microbiologically in 86% of cases, with the remaining (14%) diagnosed clinically alone. Upper and lower respiratory tract infections were noted in 25 (32%) and 12 (12.6%) cases, respectively. Bacteremia accounted for 9 (11.3%) cases, while urinary tract infection occurred in 10 (13%) instances. Notably, COVID 19 infection occurred in 10% (*n* = 7) of patients. Five cases of fungal infections were noted including candida esophagitis (*n* = 1), candida fungemia (*n* = 1) and PJP pneumonia (*n* = 3). All 3 cases of PJP occurred in patients who were not on any primary PJP prophylaxis. In this cohort, grade 1/2 infections accounted for 31 (40%) cases. Grade ≥3 infection was observed in 60% (*n* = 46) of cases including 1 grade 5 event, which occurred in the setting of severe COVID 19 infection with respiratory failure. While 2.6% of infections occurred during a hospital stay, 65% (51 events) of infections required hospitalization.

### Effects of IVIG supplement on infection

The cumulative incidence of all-grade infections at 3 and 6 months in patients on primary IVIG supplementation was 35% (95% CI, 23–55) and 35% (95% CI, 23–55), respectively. This is numerically lower than the corresponding incidence of 44% (95% CI, 33–60) and 54% (95% CI, 41–71) noted in patients not on IVIG replacement. Specifically, the cumulative incidence of ≥ grade 3 infection at 3 and 6 months in patients on IVIG supplementation was lower at 17% (95% CI, 8.7–35) each, compared to 31% (95% CI, 21–46) and 43% (95% CI, 31–61) respectively in patients not on primary IVIG prophylaxis. Further, primary IVIG supplementation resulted in a statistically significant reduction in the cumulative incidence of ≥ grade 3 infection (supplement Fig. 1). Compared to patients who did not receive IVIG supplementation, recipients of IVIG had a statistically significant reduction in rates of all grades (0.95 vs 0.45; *p* = 0.005) and ≥ grade 3 infection (0.61 vs 0.24; *p* = 0.011) per 100 days.

### Predictors of infection with teclistamab

As a last step we attempted to determine factors associated with the risk of infections. In a multivariable Cox-regression model, baseline factors such as age (HR 0.99; 95% CI 0.97,1.01; *p* = 0.33), baseline hypogammaglobinemia (HR 1.14; 95% CI 0.60, 2.19; *p* = 0.68), baseline neutropenia (HR 1.03; 95% CI 0.48, 2.18; *p* = 0.94), baseline lymphopenia (HR 0.97; 95% CI 0.54,1.75; *p* = 0.93) and prior lines of therapy (HR 1.12; 95% CI 0.95, 1.31; *p* = 0.18) were not associated with risk of infectious complications with teclistamab therapy. While cumulative number of teclistamab cycles (HR 1.09; 95% CI 0.98,1.21; *p* = 0.12), changes in absolute lymphocyte (HR 1.22 ; 95% CI 0.90, 1.67; *p* = 0.20) and absolute neutrophil counts (HR 1.0;95% CI 0.92, 1.09; *p* = 0.93), and changes in functional IgG levels (HR 1.13; 95% CI 0.97, 1.31; *p* = 0.13) were not predictive of a risk of infection, use of IVIG prophylaxis (HR 0.33; 95% CI 0.17, 0.64; *p* = 0.001) and history of infection on teclistamab therapy (HR 1.39; 95% CI 1.11, 1.74; *p* = 0.004) were independently associated with risk of infection on both univariate and multivariate analysis.

## Discussion

We report the largest real-world experience till date on the safety and efficacy of standard-of-care teclistamab in relapsed/refractory MM since its accelerated approval by the FDA. We have three key findings. First, despite a more heavily pre-treated patient population compared to the MajesTEC-1 trial with greater than two-fold higher incidence of patients with penta drug-refractory disease, the proportion of patients achieving a VGPR or better was comparable to MajesTEC-1 (51% vs 59% respectively). Second, while the overall incidence of ≥ grade 3 CRS and ICANS remained low, they were substantially higher compared to MajesTEC-1 trial, with incidences of 3.6% and 4.5% respectively (compared to 0.6% for each in MajesTEC-1). Third, the incidence of all grades and ≥grade 3 infections were lower at 40% and 26% respectively in this real-world experience, with primary IVIG prophylaxis associated with a significantly lower incidence of grade 3 or higher infections.

Our study shows that teclistamab maintains its efficacy in a heavily pre-treated cohort of patients enriched in high-risk disease, including high-risk cytogenetic abnormalities and EMD. Notably, the ≥VGPR rate for teclistamab and elranatamab in MajesTEC-1 and MagnetisMM-3 trials were 59% and 56% respectively [1, 6], which is comparable to that in our study despite a shorter follow-up, reflecting the rapidity of the onset of deep response. Of note, the lower CR rate in our cohort compared to pivotal trials is likely since bone marrow biopsy was not performed in most patients to confirm CR, and patients otherwise meeting criteria for CR but without bone marrow confirmation were labelled as VGPR. Notably, about one-third of patients in our cohort received prior BCMA-targeted therapies, majority of which were BCMA CAR T-cell therapy. However, prior exposure to BCMA-targeted therapy did not impact response rate, indicating that teclistamab can be a potential option for patients who relapse after BCMA-targeting CAR T-cell therapy, two of which are currently approved in the US. Similarly, in Cohort C of MajesTEC-1 trial that allowed prior exposure to ADCs and CAR, the % ORR among 11 patients with prior BCMA CAR exposure was 45% (17–77), with the median DOR not reached at a median follow-up of 7 months [13]. In a pooled analysis of all MAGNETISMM studies, elranatamab was able to induce a ORR of 46% and a median DOR of 17.1 months in patients previously exposed to BCMA directed therapy [14]. Nevertheless, long-term follow-up is needed to ascertain whether the durability of response with BCMA bsAbs will be similar in patients with and without prior exposure to BCMA CAR T-cell therapy.

Our results demonstrate that the risk of severe CRS and ICANS with teclistamab in the real-world setting is higher compared to that noted in clinical trials. Of note, the incidence of ≥grade 3 CRS and ICANS with teclistamab in MajesTEC-1 was 0.6% each and that with elranatamab in MagnetisMM-3 was 0% each [1, 15]. The ≥ grade 3 CRS and ICANS rate with standard-of-care teclistamab (3.6% and 4.5% respectively) is comparable to what is seen with ide-cel in real-world setting (3% and 6% respectively) [16]. Since baseline disease burden is an important predictor of severe CRS with bsAbs and CAR T-cell therapy, the greater incidence of CRS in our cohort could be explained by a higher tumor burden compared to patients enrolled in clinical trials [17]. While baseline bone marrow aspiration and biopsy were not routinely performed in our cohort, 94% had actively progressing disease at teclistamab initiation, which likely reflects a high disease burden. Future prospective studies should investigate risk-factors for severe CRS and ICANS with bsAbs, which can identify patients who would be suitable for prophylactic measures such as tocilizumab. and potentially identify patients suitable for an outpatient model of step-up dosing.

The burden of infectious toxicities remains substantial with teclistamab in the real-world setting, likely due to treatment-induced immunosuppression and high baseline tumor burden. The mechanism of immunosuppression with BCMA-targeting bsAbs include profound hypogammaglobulinemia [18, 19], T-cell exhaustion [20], and cytopenias [1, 6]. In a study on 37 patients who received BCMA bsAb monotherapy in clinical trials, 100% of responders were noted to develop severe hypogammaglobulinemia with IgG level <200 mg/dl, along with undetectable IgA and IgM level by the 2nd month of therapy [19]. Importantly, IVIG administration was associated with significantly lower infection risk in that study, with an incidence rate ratio for grade 3–5 infections being 0.1 (95% CI, 0.01-0.8; *p* = 0.0307) for periods on IVIG supplementation versus periods off-IVIG [19]. However, primary IVIG prophylaxis was not implemented in that study, with a substantial proportion of patients starting IVIG as secondary prophylaxis after a high-grade infection. To our knowledge, our study is the first to show that primary IVIG prophylaxis is associated with a significantly lower risk of all-grade infections with teclistamab, with the relative risk reduction being ~70%. In the absence of prior or ongoing RCTs testing IVIG in the setting of BCMA bsAbs, the cumulative evidence strongly suggests a substantial benefit in terms of reducing infection risk. Notably, the incidence of grade 5 infection was low (0.9%) in our study, with the caveat being short follow-up. In a prior study by our group on infections among patients enrolled in early-phase bsAb trials, the incidence of grade 5 infections with BCMA bsAb monotherapy was noted to be 8.2% at a median follow-up of ~6 months [8]. While a higher uptake of infection prophylaxis over time is a plausible explanation for reduced incidence of grade 5 infections, longer follow-up is critically important to assess the cumulative incidence of high-grade infectious toxicities with standard-of-care teclistamab since a plateau in infection risk was not observed in clinical trials [18, 19]. Notably, all cases of PJP occurred in patients who were not on prophylaxis, which is similar to our prior study, indicating the need for routine PJP prophylaxis with BCMA bsAbs in myeloma [8].

Our study has limitations. First, the follow-up is short, which makes comparison of PFS with MajesTEC-1 challenging. Second, the infection prophylaxis was heterogeneous across institutions since evidence-based guidelines were not available since teclistamab approval. Third, bone marrow aspiration and biopsy were not routinely performed for adjudication of CR and minimal residual disease (MRD) assessment. Follow-up imaging was also not routinely performed in patients with EMD at baseline. Nevertheless, the ≥VGPR rate in our cohort was comparable to what has been reported in clinical trials of BCMA bsAb monotherapy in relapsed/refractory MM. The strength of our study includes a diverse multi-institutional cohort with 39% racial/ethnic minorities and a patient population that is enriched in high-risk disease who are refractory to all active anti-myeloma agents.

In summary, our study demonstrates that teclistamab leads to rapid achievement of deep hematologic response in heavily pre-treated MM, with response rates comparable to MajesTEC-1 trial. While the incidence of severe CRS/ICANS is substantially higher in the real-world compared to what was seen in clinical trials, stringent infection prophylaxis, especially primary IVIG prophylaxis, can significantly lower infection-related morbidity and mortality. Future studies should prospectively assess the impact of primary IVIG prophylaxis along with limited-duration treatment to potentially avoid T-cell exhaustion and allow safer delivery of BCMA bsAbs.

### Supplementary information

Supplementary Table 1 and 2

Supplementary Figure 1

## Data Availability

The datasets generated during and/or analyzed during the current study are available from the corresponding author on reasonable request.
