# Supplementary material for: Teclistamab in relapsed refractory multiple myeloma: multi-institutional real-world study
Source: Blood Cancer J. 2024 Mar 5;14(1):35. doi: 10.1038/s41408-024-01003-z (PMC10914756; doi:10.1038/s41408-024-01003-z)
Supplement: Supplementary file 1 — Supplementary Table 1 and 2 [file 41408_2024_1003_MOESM1_ESM.docx]

**Supplement Table 1: Baseline Characteristics**

| **Variables** | Median (Range); n (%)  N=110 |
| --- | --- |
| Median Age | 68 (37 - 89) |
| Female | 54 (49%) |
| **Race** | |
| Asian/Pacific Islander | 2 (1.8%) |
| Black | 32 (29%) |
| Other | 9 (8.2%) |
| White | 67 (61%) |
| **Ethnicity** | |
| Hispanic | 11 (10%) |
| Non-Hispanic | 99 (90%) |
| **Heavy chain subtype** | |
| IgA | 35 (33%) |
| IgG | 54 (51%) |
| IgM | 1 (0.9%) |
| Other | 16 (15%) |
| Unknown | 4 |
| **Light chain subtype** | |
| Kappa | 77 (70%) |
| Lambda | 33 (30%) |
| **FISH studies** |  |
| t (11;14) ^¡^ | 19 |
| t (4;14) ^¥^ | 15 |
| t (14;16) ^±^ | 10 |
| t (14;20) ^£^ | 2 |
| Deletion 17p^∞^ | 23 |
| 1q21 copy number alterations (3 copies) ^$^ | 42 |
| 1q21 copy number alterations (≥ copies) ^#^ | 16 |
| Deletion 1p^*^ | 15 |
| High risk disease | 59 (62%) |
| EMD | 48 (44%) |
| Number of prior lines of therapy | 6 (3-13) |
| Triple class refractory disease | 95 (86%) |
| Penta drug refractory disease | 84 (76%) |
| Recipient of ≥ 1 prior ASCT | 86 (87%) |
| Serum creatinine (mg/dl) | 1 (0.49-10.35) |
| Median Absolute neutrophil count (x 10^3^/µl) | 2.74 (1.75-3.79) |
| Median Absolute lymphocyte count (x 10^3^/µl) | 0.82 (0.47-1.29) |
| Hypogammaglobinemia | 32 (32%)^ |
| Functional hypogammaglobinemia | 37 (39%)× |
| Exposure to prior BCMA directed therapy | 38 (35%) |
| Recipients of prior BCMA CAR T cell therapy | 18 |
| Recipients of prior investigational BCMA bsAb | 5 |
| Outpatient teclistamab step-up dosing | 10 (9.1%) |

¡ Data missing in 13 patients; ¥ data missing in 14 patients; ± data is missing in 14 patients; £ data is missing in 14 patients.

∞ data is missing in 13 patients, $ data is missing in 15 patients; # data is missing in 15 patients; * data is missing in 14 patients.

^ IgG levels were missing in 9 patients: × Data missing in 16 patients

FISH: fluorescence in situ hybridization studies; EMD: extramedullary disease; ASCT: autologous stem cell transplant.

BCMA: B cell maturation antigen; bsAb: bispecific antibody.

**Supplement Table 2: Overall response rates to teclistamab therapy across different subgroups**

| **Subgroups** | **ORR (no)** | **ORR (yes)** |  |
| --- | --- | --- | --- |
| **High risk disease** |  |  | 0.42*^2^* |
| FALSE | 12 (35%) | 22 (65%) |  |
| TRUE | 22 (44%) | 28 (56%) |  |
| Missing data | 3 | 11 |  |
| **EMD on PET or MRI** |  |  | 0.46*^2^* |
| N | 20 (35%) | 37 (65%) |  |
| Y | 17 (43%) | 23 (58%) |  |
| Missing data | 0 | 1 |  |
| **Prior BCMA-Targeted Therapy** |  |  | 0.23*^2^* |
| N | 21 (33%) | 42 (67%) |  |
| Y | 16 (46%) | 19 (54%) |  |

ORR: overall response rates; High risk disease was defined as the presence of t(4;14); t(14;16); t(14;20); 1q21 copy number abnormalities, or deletion (17 p) by FISH; EMD: extramedullary disease; ASCT: autologous stem cell transplant. BCMA: B cell maturation antigen; bsAb: bispecific antibody.

**Supplement Figure 1:** (A) The cumulative incidence of all grades and ≥grade 3 infections with and without IVIG supplements in patients on teclistamab.
