# Supplementary figures and images for: Teclistamab in relapsed refractory multiple myeloma: multi-institutional real-world study
Source: Blood Cancer J. 2024 Mar 5;14(1):35. doi: 10.1038/s41408-024-01003-z (PMC10914756; doi:10.1038/s41408-024-01003-z)

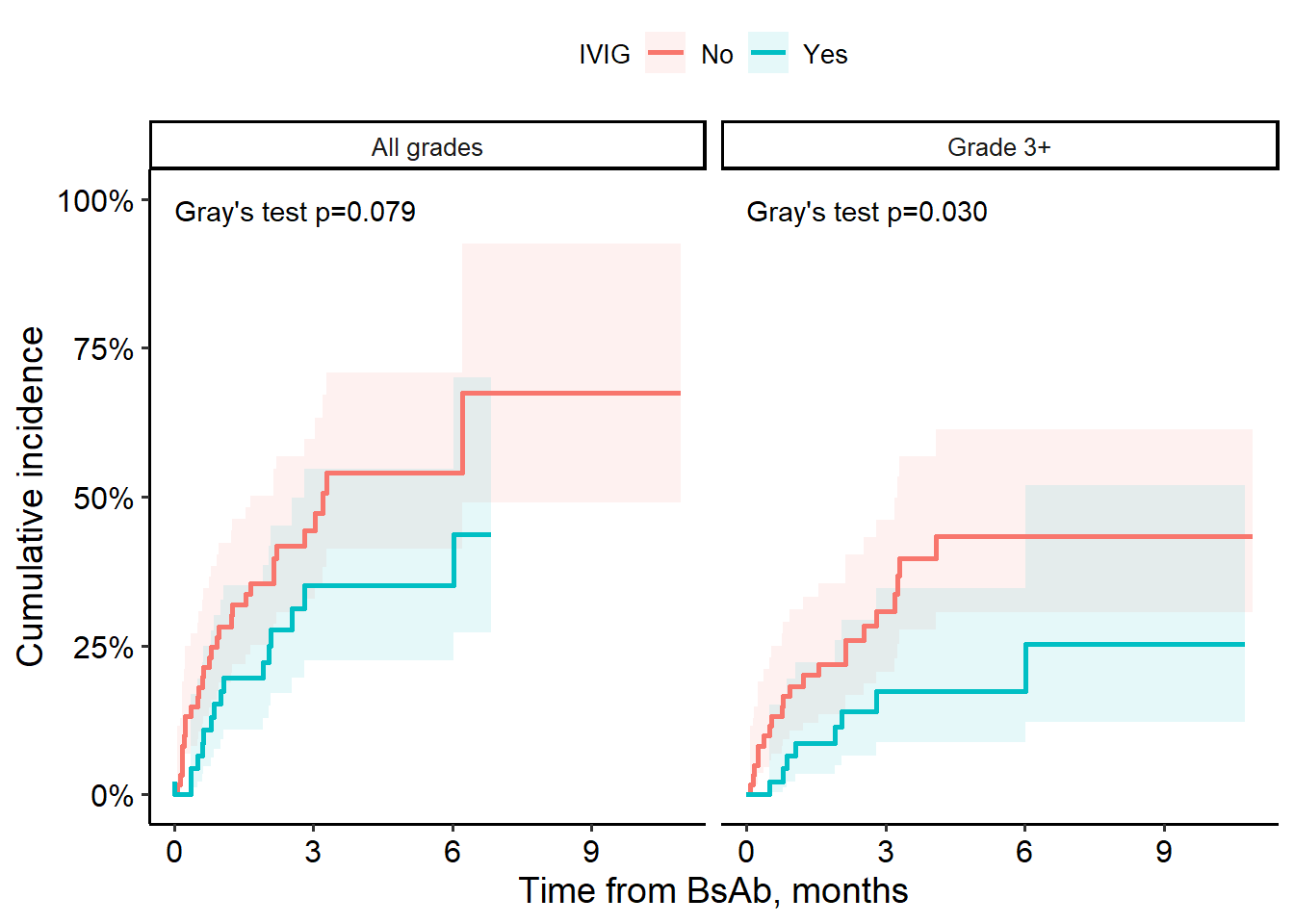

Supplement: Supplementary file 2 — Supplementary Figure 1 [file 41408_2024_1003_MOESM2_ESM.png]
